# Integrating multiple types of data to predict novel cell cycle-related genes

**DOI:** 10.1186/1752-0509-5-S1-S9

**Published:** 2011-06-20

**Authors:** Lin Wang, Lin Hou, Minping Qian, Fangting Li, Minghua Deng

**Affiliations:** 1 Center for Theoretical Biology, Peking University, Beijing 100871, China; 2 LMAM, School of Mathematical Sciences, Peking University, Beijing 100871, China; 3 Center for Statistical Science, Peking University, Beijing 100871, China; 4 School of Physics, Peking University, Beijing 100871, China

## Abstract

**Background:**

Cellular functions depend on genetic, physical and other types of interactions. As such, derived interaction networks can be utilized to discover novel genes involved in specific biological processes. Epistatic Miniarray Profile, or E-MAP, which is an experimental platform that measures genetic interactions on a genome-wide scale, has successfully recovered known pathways and revealed novel protein complexes in *Saccharomyces cerevisiae* (budding yeast).

**Results:**

By combining E-MAP data with co-expression data, we first predicted a potential cell cycle related gene set. Using Gene Ontology (GO) function annotation as a benchmark, we demonstrated that the prediction by combining microarray and E-MAP data is generally >50% more accurate in identifying co-functional gene pairs than the prediction using either data source alone. We also used transcription factor (TF)–DNA binding data (Chip-chip) and protein phosphorylation data to construct a local cell cycle regulation network based on potential cell cycle related gene set we predicted. Finally, based on the E-MAP screening with 48 cell cycle genes crossing 1536 library strains, we predicted four unknown genes (*YPL158C*, *YPR174C*, *YJR054W*, and *YPR045C*) as potential cell cycle genes, and analyzed them in detail.

**Conclusion:**

By integrating E-MAP and DNA microarray data, potential cell cycle-related genes were detected in budding yeast. This integrative method significantly improves the reliability of identifying co-functional gene pairs. In addition, the reconstructed network sheds light on both the function of known and predicted genes in the cell cycle process. Finally, our strategy can be applied to other biological processes and species, given the availability of relevant data.

## Background

According to [1], “mutations in two genes produce a phenotype that is surprising in light of each mutation's individual effects. This phenomenon, which defines genetic interaction, can reveal functional relationships between genes and pathways.” Thus, deciphering genetic interaction networks via high-throughput technologies can both reveal the schematic wiring of biological processes and predict novel genes. Recently, several such high-throughput technologies have been developed to identify genetic interactions at the genome scale, including Synthetic Genetic Array (SGA) [2], Diploid-based Synthetic Lethality Analysis on Microarrays (dSLAM) [3], and Epistatic Miniarray Profile (E-MAP) [4]. The first two approaches aim to identify synthetic lethal, or negative, interactions, meaning that the double mutant is more lethal than the corresponding single mutants. On the other hand, assuming that the expected phenotype of a double mutation reflects the additional effects of the single mutations, E-MAP, an extension of SGA, gains power by identifying positive as well as negative interactions, which, in this case, would indicate that the double mutant is healthier than expected.

Here, we exploited the E-MAP methodology to discover novel genes involved in the cell cycle process in budding yeast. The distinct advantage of using E-MAP is the potential of discovering functionally associated genes which do not otherwise physically interact. Physical interaction assays, such as the yeast two-hybrid system or DNA-binding microarrays, are unlikely to reveal these associations. Despite the superiority of E-MAP, interpretation of the data is still challenging. First, genetic interactions occur both between and within functional modules. Thus, the function of a gene cannot be determined by its interacting partners. Second, E-MAP suffers from high false positive and negative rates, making it difficult to infer genetic interaction accurately and sufficiently. Consequently, the integration of external information, such as gene expression, Transcription Factor (TF)-DNA binding (chip-chip) and protein phosphorylation, is necessary in order to identify novel genes involved in the cell cycle process.

Several methods have been developed to integrate multiple types of data to infer a transcription regulatory network in eQTL analysis, including mRNA expression, chip-chip, physical interaction and protein phosphorylation [5-8]. In this paper, we integrated genetic interaction and other genomic data to construct a specific network which we then applied to the cell cycle process in budding yeast.

## Results

### Construct a potential cell cycle-related gene set

**Figure 1:**
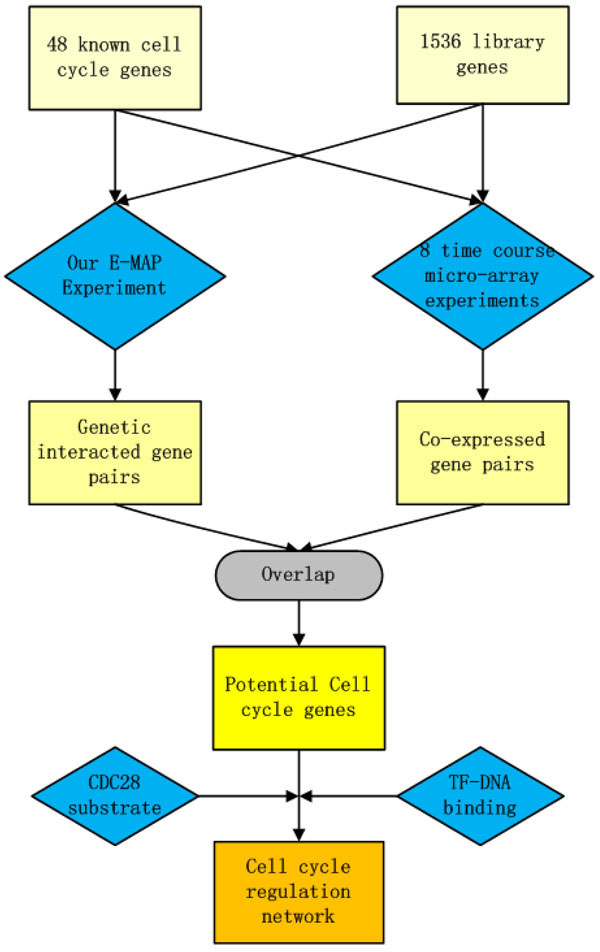
Overview of our strategy to integrate multiple types of data to identify cell cycle gene and infer regulatory pathways.

As indicated in Figure 1, our strategy, which integrates multiple types of data, aims to include all potential cell cycle genes within the known cell cycle gene set. Since both genetic interacting and co-expressed gene pairs tend to be co-functional, we hypothesized that a potential cell cycle related gene set with higher confidence can be achieved through combining the two data sources, compared with using either data alone.

**Figure 2:**
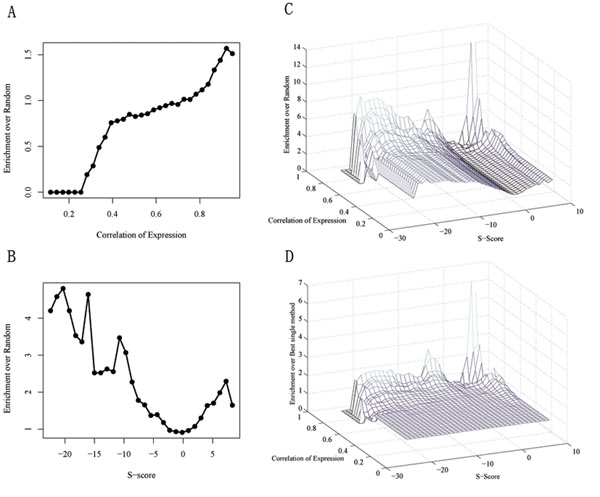
**Combining genetic interaction and co-expression data to define co-functional gene pairs**. High correlation coefficient (A) and extreme S-Score both correspond with known co-functional gene pairs in Gene Ontology (GO) (bin size is 1 Correlation and 1.5 for S-Score). (C) Gene pairs have more extreme S-score and larger correlation of expression have higher enrichment. (D) When combining two criteria, the enrichment is significantly improved in extreme S-score and large correlation area.

To accomplish this, we first quantitatively measure whether genetic interactions and co-expression indicate co-functional membership and, if so, to what degree. The E-MAP method was adopted for genetic interaction analysis. Forty-eight known cell-cycle genes (KCCGs, Table S1 in Additional file 1) were screened against a library of 1536 test strains in budding yeast, yielding a quantitative value (S-Score defined in [3]) for 67680 gene pairs (91% of all possible pair-wise measurements). Co-expressed data were then calculated from 8 groups of time-course expression datasets generated in four previously published studies (see Methods and materials for details). To calculate the enrichment of co-functional gene pairs over random gene pairs, we first compute the fraction f of interactions at each S-score (or cc, correlation coefficient of expression) (Figure 2A and 2B) or simultaneously more extreme than s and cc (Figure 2C and 2D) that fall inside one biological process term in GO for certain bin sizes. Then the enrichment is the ratio f/r, where r is the fraction of random gene pairs which participate in the same GO biological processes.

As expected, Figure 2A confirms that gene pairs having a higher cc are more likely to be co-functional. Also, Figure 2B shows that gene pairs with both significant positive and negative S-scores are more likely to be co-functional. By comparing the enrichment between Figures 2A and 2B, it is apparent that extreme S-scores could indicate co-functional membership more efficiently.

When combining these two kinds of data, we found that they were complementary. As shown in Figure 2C, for a certain cut-off of S-scores, gene pairs with a higher correlation of expression are more likely to be co-functional, and vice versa. Therefore, the results approved our original hypothesis that combining these two kinds of information could help to construct a more accurate potential cell cycle related gene set. We adopted an area by which to define significantly interacting gene pairs based on the data in Figure 2C. For a positive genetic interaction area, we require that the enrichment over random be larger than 2, and for a negative genetic interaction area, we require that it be larger than 4. Then the constraints are (S-score>2.5 and cc >0.9) or S-score>6 or (S-score<-3 and cc>0.9) or (S-Score<-14 and cc>0.85). Compared to the most powerful method at each point, the combination is generally >50% more accurate in the areas defined above (Figure 2D). Finally, 259 gene pairs between 206/1536 test strains and 48 KCCGs passed the filter. We use these 206 test genes as the potential set of cell cycle-related genes (PCCGs).

### Recovery of known genetic interactions with our E-MAP

We compared our E-MAP data with the benchmark data. Similar to previous work [9], we tested the sensitivity and precision of the E-MAP data (see Methods and materials). Compared to genetic interactions in BIOGRID, both the positive and negative interactions are very precise (p-value < 10^–50^).

We also tested the efficiency when combining E-MAP with DNA microarray data. When the co-expression test was applied, the significance level of precision increases around 2-fold (Table S2 in Additional file 2). This result indicates that co-expression does indeed provide extra information about genetic interaction. Hence, our strategy can be used to identify potential cell cycle genes and their relationships with known cell cycle genes, thus enabling us to construct a reliable network.

We also compared our S-score with previously published large-scale genetic interaction data [8]. Significantly interacting gene pairs show obvious correlation between the two datasets (r=0.64, Figure S1 in Additional file 3).

### Functional enrichment analysis in the PCCGs

Functional enrichment analysis was performed on all GO biological process terms in both positive and negative parts of PCCGs. We defined the positive part as those genes having (S-score>2.5 and cc >0.9) or S-score>6 and the negative part as those genes having (S-score<-3 and cc>0.9) or (S-Score<-14 and cc>0.85). We distinguished these two parts because the principles on which positive and negative genetic interaction are based may be different for functional analysis, as discussed in a previous study [10]. For the positive part, only one functional category, “nucleosome organization,” was enriched under 98% confidence level (q=0.012). For the negative part, five functional categories were enriched, including “DNA-dependent DNA replication,” “chromatin assembly,” “interphase of mitotic cell cycle,” “cell cycle checkpoint” and “regulation of organelle organization” (q=0.014, 0.013, 0.015, 0.009, 0.012). All these biological processes can either be interpreted as related to the cell cycle process, or just part of it. In addition, KCCGs were found to be mainly involved in “regulation of organelle organization,” “regulation of mitotic cell cycle,” “interphase of mitotic cell cycle,” “regulation of cell cycle process” and “cell cycle checkpoint” processes. Hence, in the negative part, there are more directly co-functional genes than the positive part. This is consistent with the surprising conclusion of previous work [9] indicating that negative, in contrast to positive, genetic interactions always occur between genes with overlapping functions.

Finally, we also analyzed the functional enrichment of all PCCGs. Three functions, including “chromatin assembly,” “regulation of organelle organization” and “nucleosome organization,” were enriched (q=0.015, 0.009, 0.002). This suggests the importance of separating PCCGs into two parts for a functional enrichment analysis. Such separation further helps us to understand how known cell cycle genes, both positive and negative, interact in terms of their functions and also helps us to find specific functions only enriched in one of the two parts, such as “DNA-dependent DNA replication,” “interphase of mitotic cell cycle” and “cell cycle checkpoint.” which only enriched in negative part but not in all PCCGs.

### A cell-cycle transcriptional network based on the PCCGs and KCCGs

**Figure 3:**
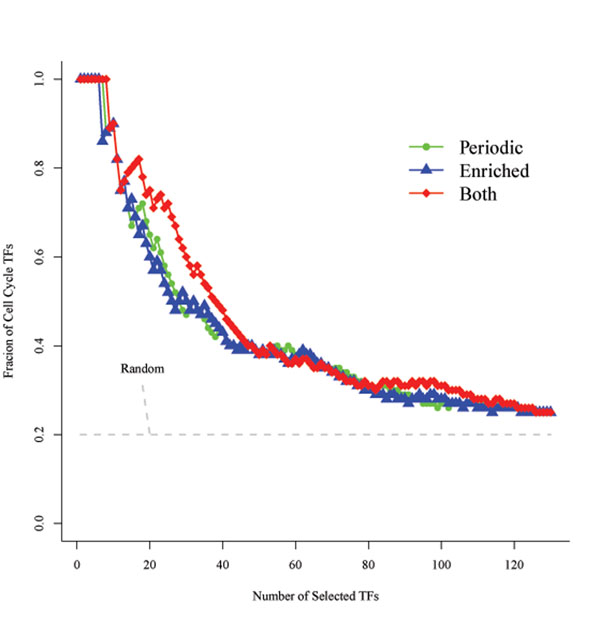
**Comparison of the fraction of cell cycle TFs selected by different standards**. Comparison of the fraction of cell cycle TFs selected by different standards: enriched rank, periodic rank, and their combination (the rank of multiplication of the two ranks). All three methods we adopted tend to select more cell cycle TFs than random selection (Fraction=37/183=0.2). The enriched test (ET) and periodic test (PT) show similar power, while ET and PT in combination can increase the power.

In the next step, we searched for main transcription factors (TFs) regulating both the PCCGs and the KCCGs based on TF-DNA binding data (Chip-Chip), and then we constructed the resulting transcriptional regulatory network. In previous studies, Chip-Chip data are usually combined with expression information to construct the regulatory network. In our method, periodic expression was required for TF inclusion. Since most genes involved in the cell cycle process are expressed periodically, it is reasonable to assume corresponding periodicity of their transcriptional regulators. In addition, we also assumed that the regulatory targets of a TF involved in the cell cycle would be enriched for the known cell cycle gene. Hence, our transcription network was based on TFs enriched for cell cycle targets for both potential and known cell cycle genes combined from the pool of PCCGs and KCCGs. The significance of periodicity and enrichment of cell cycle genes can be calculated (see Methods and materials). Both approaches tend to select TFs which are known to be involved in cell cycle regulation according to MIPS functional annotation (Figure 3[11]).

Periodicity and enrichment are consistent criteria since most of the known cell cycle TFs rank at the top in both cases. However, some TFs are ranked differently (See Table S3 in Additional file 4). For example, *Mcm1* is ranked 6/130 in the enrichment test (ET); however, it ranks 124/183 in the periodic test (PT), which means that its expression does not show periodicity. We know that *Mcm1* regulates different phases during the cell cycle [12,13], and its expression will not be periodic. However, many of its neighboring genes in the transcriptional network are cell cycle genes, making its identification possible in the enrichment test. Similar to *Mcm1*, *Skn7* ranks 23 and 114, respectively, in ET and PT. In contrast, *HCM1* is ranked 3 in PT, but 42 in ET. One possible explanation of this apparent difference is that the PCCGs and KCCGs only cover a limited part of cell cycle-related genes, and some targets of *HCM1* are missing in this set. Other examples like *YHP1* are similar to *HCM1*. Based on this analysis, a TF that is significant in either test should be included. Hence, we use the multiplication of the two ranks as an index, and we use its rank to evaluate the priority order (see Methods and materials for details).

To determine how many TFs should be involved, we examined the coverage rates of TFs. The coverage rate is evaluated at two levels: the fraction of genes which are targets of the selected TFs in the PCCGs and KCCGs and the fraction of gene pairs which are co-regulated by any one of the selected TFs. In the PCCGs and KCCGs, 232/236 genes are involved in the chip-chip dataset (at least one TF can bind to them), and 77 gene pairs, which are both genetically interacting and co-expressed, can be simultaneously bound by the same TF. We noticed that when the top 25 TFs are selected, most of the 232 genes and 77 gene pairs (75% and 97%, respectively) could be covered (Figure S2 in Additional file 5). The cover rate increases quite slowly when more TFs are selected. We therefore used these 25 TFs to construct the transcriptional network based on the PCCGs and KCCGs.

### Enrichment for cell-cycle genes and TFs

We next determined whether the PCCGs are enriched with known cell-cycle genes. Among the PCCGs, we calculated the proportion of genes which are annotated to participate in the cell cycle process (in MIPS database) and used the hyper-geometric distribution to define the p-values. About 1/2 of the PCCGs (94/206) were determined to be known cell-cycle and DNA processing genes (*p* = 6 × 10^–6^). We performed the same test to the selected TFs. Eighteen of them are known to be cell cycle TFs (*p* = 5 × 10^–11^, Table S4 in Additional file 6).

### Enrichment for *CDC28* substrates

Since cell cycle events are controlled by cyclin-dependent kinases (CDKs), we investigated whether Cdk1(*CDC28*) substrates were enriched in our PCCGs and selected TFs. As expected, both of them turned out to be enriched with *CDC28* substrates (Table S5 in Additional file 7), further supporting the finding that both PCCGs and selected TFs are cell cycle-related.

### Formation of a cooperative transcriptional network by selected TFs is supported by indirect evidence

We compared the difference between using all TFs in the database and only the selected 25 TFs to explain indirect transcriptional relationships between the 25 TFs and 232 target genes. Based on comparing the wild-type and TF mutant microarray data, we could tell how one TF could affect the expression of each gene. This data reflects the transcriptional relationship between the TFs and targets although the relation could be indirect. This independent evidence, which describes the transcriptional network, can be utilized to validate the network we constructed.

Between our 25 TFs and the 232 target genes, there are 140 indirect TF-target pairs. By using the transcriptional relationships of all 183 TFs in the chip-chip dataset, 103/140 pairs could be connected within three steps, although for more steps, quite a few indirect pairs could be explained (Figure S3 in Additional file 8). We also tested the fraction of indirect TF-target pairs which could be connected by only using the relationships of the 25 TFs. The result (Figure S3) shows that the sub TF-target network can explain 85.4% (88/103) of the indirect relations in the first three steps. This result illustrates that the 25 TFs form a cooperative transcriptional network which can explain its indirect connections quite well.

### Clustering of the constructed transcriptional network

**Figure 4:**
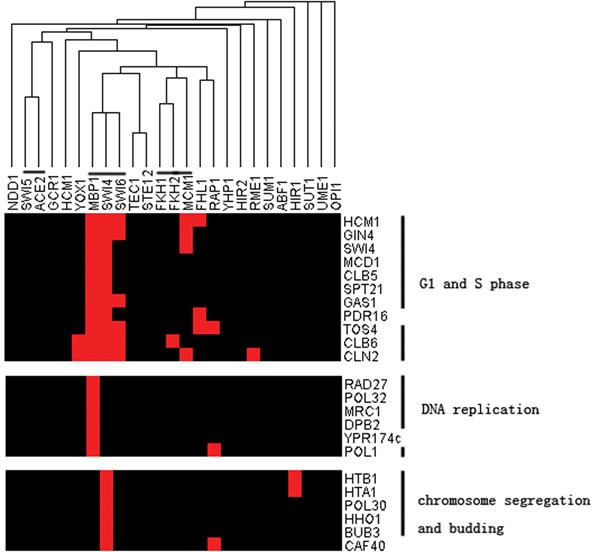
**Three clusters in the transcriptional network**. Red box indicates the corresponding TF regulates the gene in chip-chip data. For TFs (above); we have lined out three known cooperative TF clusters; for the three gene clusters (right), we lined out genes which have similar functions in the cluster.

To understand the structure of our constructed transcriptional network, we used the transcriptional profile to do cluster analysis (Figure S4 in Additional file 9). That TFs with similar function regulate similar targets in the network can be inferred by the presence of several established cooperating TF clusters, such as *Ace2/Swi5*, *Fkh1/Fkh2/Mcm1* and *Swi4/Swi6/Mbp1*. In addition, genes with similar function can also cluster together and have meaningful explanation in the context of their TFs. Figure 4 shows three such functional clusters. The first one contains G1/S and S phase functional genes which are simultaneously regulated by important G1/S transcriptional regulators, SBF (*Swi4/Swi6*) and MBF (*Mbp1/Swi6*). The second contains DNA replication-related genes which are mainly regulated by *Mbp1*. The third contains chromosome segregation- and budding-related genes which are mainly regulated by *Swi4*. These results are consistent with our knowledge about the function of SBF and MBF in the cell-cycle process. Hence, the structure of our transcriptional network revealed by the cluster analysis could also be used to infer functional relationships between genes.

### Potential cell cycle-related genes

**Figure 5:**
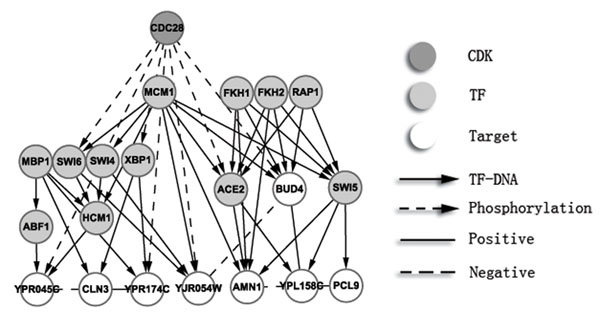
**One network involves four potential cell cycle-related genes with unknown function**.

**Figure 6:**
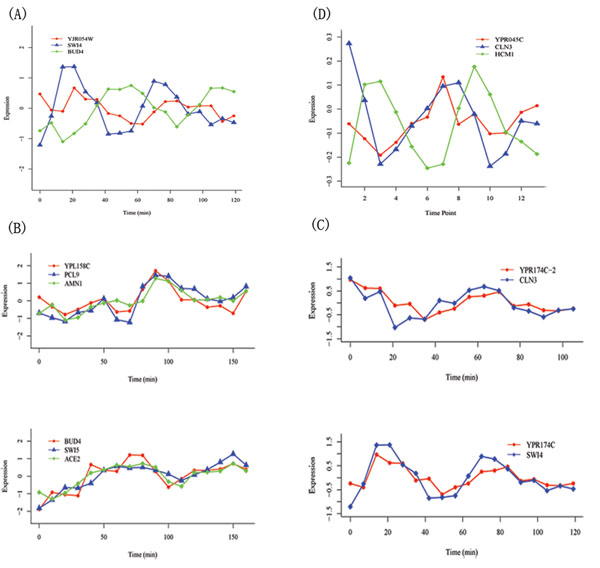
**Expression data analysis for the four cell cycle related genes we predicted**. (A) The expression of YJR054W, SWI4 and BUD4 in Spellman-alpha experiment. SWI4 and BUD4 are significantly negatively correlated (upper), and YJR054W looks similar to SWI4, although it is appropriately one time point lagged. (B) The expression of YPL158C related genes in Cho-cdc28 experiment. The genes can be separated into two groups. The first group includes YPL158C, PCL9 and AMN1 (upper), and the others form the second group(below). Genes in each group are co-expressed, and the peak value of the first group is lagged comparing to the second one. (C) The expression of YPR174C, CLN3 and SWI4 in Spellman-alpha experiment. YPR174C is co-expressed with SWI4. YPR174C is left shifted for 2 time points (Noted as YPR174C-2), and it’s also co-expressed with CLN3. (D) The expression of YPR045C, CLN3 and HCM1 in Pramila-alpha26 experiment. YPR045C and CLN3 are co-expressed, while HCM1 is negative correlated with them and is slightly earlier than them.

Based on E-MAP, expression, chip-chip, and protein phosphorelation data and the analysis above, we could identify PCCGs and know about its structure in transcriptional network (results are summarized in Table S6 in Additional file 10), Among the PCCGs, we will introduce four genes with unknown function (Figure 5).The first one is *YPL158C*, which genetically interacts with *PCL9*, *AMN1* and *BUD4*. These four genes are all regulated by known TFs in M phase (including G2/M and M/G1). The expression data show that *YPL158C*, *PCL9* and *AMN1* are simultaneously expressed and that their peak value of expression is later than that of *ACE2* and *SWI5*, which are their transcriptional regulators, as well as *BUD4* (Figure 6A). This is consistent with the regulatory network because *BUD4*, *ACE2*, and *SWI5* are mainly regulated by *FKH1/2* and *MCM1*, while *YPL158C* is mainly regulated by *ACE2* and *SWI5*. They all act in M phase or early G1 phase. Based on these observations, *YPL158C* is possibly involved in M phase and co-operates with *PCL9*, *AMN1* and *BUD4*. The second is *YPR174C*, which genetically interacts with *CLN3* and potential substrates of *CDC28*. *YPR174C* and *CLN3* are co-regulated by *MBP1* and *XBP1*, another known cell cycle TF, which was not selected in the procedure above because of low periodic rank (rank in PT: 120; rank in ET: 11; final rank: 34). According to the description in SGA, *XBP1* is a member of the *SWI4/MBP1* family. Since *MBP1* and *XBP1* do not have significant periodic expression, we compared the expression of *SWI4* with *CLN3* and *YPR174C*. We found that *SWI4* and *YPR174C* are significantly co-expressed and that *CLN3* and *YPR174C* also show a co-expression pattern, but with two time points lagging (Figure 6B). It is convincing to consider that *YPR174C* might be involved in G1 phase in cell cycle process since all other related genes are mainly acting in this phase. In addition, based on the transcriptional analysis above, *YPR174C* is mainly regulated by *MBP1*; hence, it is possibly involved in the DNA replication process. The third one is *YJR054W*, which genetically interacts with *BUD4* and potential substrates of *CDC28*. In Chip-Chip data, *BUD4* is regulated by *MCM1*. *YJR054W* is regulated by *SWI4* and *SWI6* which are also regulated by *MCM1*. In the expression data (Figure 6C), we found that the expressions of *SWI4* and *BUD4* are highly negatively correlated. This can be explained by the fact that *MCM1* participates in the formation of both repressor and activator complexes, and *SWI4* and *BUD4* may be regulated by different complexes. The expression of *YJR054W* is similar to *SWI4* and slightly lags, which supports the regulation between them. Since *BUD4* can influence the next round of budding and *SWI4/6* mainly regulates the G1 phase, *YJR054W* may be involved in M/G1 phase and may co-operate with *BUD4*. The last one is *YPR045C*, which genetically interacts with *CLN3*, albeit negatively, and potential substrates of *CDC28*. It is regulated by *HCM1* and *ABF1* which both regulate S-phase during the cell cycle process. *YPR045C* is negatively correlated with *HCM1* (Figure 6D), which may suggest that *HCM1* suppresses the expression of *YPR045C*. Considering that *CLN3* is G1 cyclin and activates *Cdc28* kinase to promote the G1 to S phase transition, we suggest that *YPR045C* could play a role during G1 and S phase.

## Discussion

Our approach integrates the genetic interaction network, co-expression network, and transcriptional network, and it performed well in predicting cell cycle genes. Many previous papers have also discussing integrating these data source in eQTL analysis. However, comparing to these approaches, we started from known functional genes and their E-MAP profiles to build up the network step by step. In this process, we could see how these data source could describe the biology network, and how they are co-operated together. We illustrate that E-MAP and DNA microarray could be complementary in identifying PCCGs, and also the cluster result tells that how transcriptional relationships could reflect functional connections of genes in the network.

In addition, there are other types of networks, such as protein physical interaction networks, which are informative for the prediction of gene function. However, because physical interactions annotated in databases are quite sparse between KCCGs and the 1536 library strains, we have not performed an analysis of it. We believe the efficiency of prediction can be increased when such data are integrated in a reasonable framework.

Although the current study focused on the cell cycle process, our approach is not limited, and it can be easily applied to other biological processes, given the availability of data.

## Conclusions

E-MAP technology is a powerful high-throughput tool to identify novel functional genes which genetic interacted with the known one. By screening forty eight cell cycle genes crossing 1536 library strains, E-MAP helps us obtain a large potential cell cycle-related gene set.

Our analysis shows that genetic interaction and gene co-expression could be complementary for identifying co-functional gene pairs, and combining them has significantly improved the accuracy of the prediction.

TF-DNA binding (chip-chip) and protein phosphorylation data were used to construct a cell cycle regulation network. Periodic expressed and being enriched of cell cycle genes in targets can both be used to identify TFs which regulate the cell cycle process. When comparing the cluster result to prior knowledge, we could believe that our cell cycle transcriptional network is well constructed. This network could help to illustrate how PCCGs are involved in cell cycle process.

Finally, four genes with unknown functions in PCCGs are laboured. From KCCGs which the four genes are genetic interacted and co-expressed, we could predict which phase of cell cycle they may be involved in. In addition, the time course expression data of them are consistent with the constructed transcriptional network, and some of them are substrate of CDK1 (*CDC28*) kinase which regulates the cell cycle process in budding yeast. All these analyses provided strong evidence that the four novel genes should be participate in cell cycle related process.

## Methods and materials

### E-MAP experiment data

The 48 cell cycle genes were manually curate from the literature, which function in different phases of the cell cycle process (Figure S5 in Additional file 11). They had been screened by crossing a 1536 mutant strain library in budding yeast, and the relative double mutant strains were selected to obtain the EMAP data. However, the analytical framework we developed is not affected by the selection of these genes, and it can be applied to other processes as well.

### Time course expression data and definition of correlation

We use eight time course microarray experiments from four previously published works to perform the co-expression analysis [14-17]. The data were downloaded from the supplementary data from the authors’ website, and the KNNImpute method was used [18] to impute the missing value.

To measure the similarity between the time course expression profiles of two genes, we used the time-lagged correlation [19]. For multiple experiments, we adopted a loose definition for correlation between two genes which is the maximum time lag correlation score in all the eight experiments. This means that two genes showing high correlation in one experiment are considered to be co-expressed. We can use such a loose definition because we have already had a stringent constraint in E-MAP analysis to ensure that the interactions are reliable, even if two genes only show co-expression in one experiment.

### Comparison with SGA genetic interaction data

The true genetic interactions we used here are previously published interactions from Biogrid (http://www.thebiogrid.org). Similar to previous work, for negative genetic interactions, we also considered interactions annotated as phenotypic enhancement, synthetic growth defect and synthetic lethality. For positive genetic interactions, interactions annotated as phenotypic suppression and synthetic rescue are used. By using S-score cut-offs, we calculated the number of true positives (TP) as the number of Biogrid interactions with S-scores more extreme than the cut-offs. As defined in previously published work, sensitivity is defined as the fraction of known interactions.

Precision is defined as the fraction of true interactions in the set of all predicted pairs.

We also use hyper geometric distribution to calculate the p-value of precision. The relative results are reported in Table S2 in Additional file 2.

### Definition of p-values of enrichment analysis for biological process terms in GO

We also use hyper geometric distribution to calculate p-value to measure the enrichment of one biological process in GO annotation as follows:

Here *m_i_* is the number of selected genes which have the function i; *n* is the number of selected genes; *M_i_* is the number of test genes which have the function i; N is the number of test genes.

Finally, we use Bonferroni correction to control false discovery rate in this multiple testing problem and get the q value.

### Transcriptional Regulation and *CDC28* substrate datasets

The Chip-Chip data and wild type vs. TF mutant microarray data were downloaded from YeastRact (http://www.yeastract.com[20,21]). Among 183 TFs in our dataset, 37 are annotated as cell cycle-related in the MIPS database. The CDC28 substrate dataset was downloaded from the supplementary data of two previously published works [22,23].

### Definition of p-values for periodicity and enrichment for cell cycle genes

The significance of periodicity was previously defined [24,25]. The data were downloaded from http://www.cyclebase.org. We also used the hyper geometric distribution to calculate the p-value for the enrichment of cell cycle targets.

Here, *m_i_* is the neighbor in the PCCGs and KCCGs; *n* is the number of genes in PCCGs and KCCGs; *M_i_* is*TF_i_*’s targets in the test genes, and *N* is the number of test genes.

### Multiplication of ranks can represent “or” relationship between the two methods

Suppose the probability of one *TF_i_* not to be enriched and periodically expressed is . Then the probability that *TF_i_* is either enriched or periodically expressed is. . For*TF_i_*, the multiplication of its rank of p-values (ascending order)  keeps the order of the probability; . Thus the smaller the order of  is, the larger *Pr_i_* of *TF_i_* is.

## Competing interests

The authors declare that they have no competing interests.

## Authors' contributions

MD and FTL initiated the study, LW conceived the study and performed the statistical analysis; LH participated in the study and algorithm design and helped to draft the manuscript. All authors read and approved the final manuscript.

## Supplementary Material

Additional file 1 **48 cell cycle query genes** This file can be viewed with Microsoft Excel Viewer. Click here for file

Additional file 2 **Sensitivity and precision of E-MAP genetic interaction scores** This file can be viewed with Microsoft Excel Viewer. Click here for file

Additional file 3 **Correlation between our E-MAP data and published data** This file can be viewed with Adobe Reader. Click here for file

Additional file 4 **Ranks of p-values for top 25 TFs** This file can be viewed with Microsoft Excel Viewer. Click here for file

Additional file 5 **Cover rate when different TFs are selected** This file can be viewed with Adobe Reader. Click here for file

Additional file 6 **Cell cycle genes are enriched in the PCCGs and TFs** This file can be viewed with Microsoft Excel Viewer. Click here for file

Additional file 7 CDC28 substrates are enriched in the PCCGs and TFs Click here for file

Additional file 8 **Indirect TF-Target connection analysis** This file can be viewed with Adobe Reader. Click here for file

Additional file 9 **Cluster results of transcriptional network** This file can be viewed with Adobe Reader. This file can be viewed with Microsoft Excel Viewer. Click here for file

Additional file 10 **Summary of our analytical results** This file can be viewed with Microsoft Excel Viewer. Click here for file

Additional file 11 **Composition of the signaling E-MAP** This file can be viewed with Adobe Reader. Click here for file
