# Supplementary material for: Integrating multiple types of data to predict novel cell cycle-related genes
Source: BMC Syst Biol. 2011 Jun 20;5(Suppl 1):S9. doi: 10.1186/1752-0509-5-S1-S9 (PMC3121125; doi:10.1186/1752-0509-5-S1-S9)
Supplement: Additional file 3 — Correlation between our E-MAP data and published data This file can be viewed with Adobe Reader. [file 1752-0509-5-S1-S9-S3.pdf]

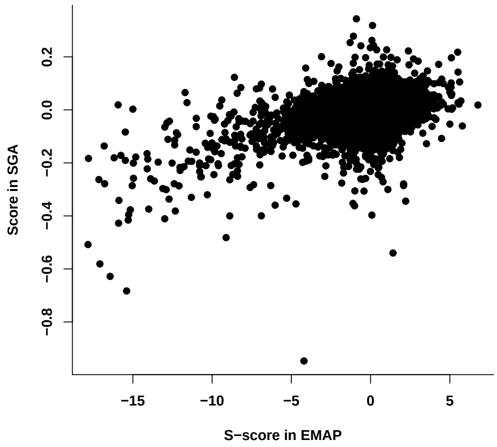

Figure S1 Correlation between our E-MAP data and published data. The interaction of 12709/ 67680 gene pairs in our E-MAP profile are also measured in [9]. The figure shows the correlation between the genetic interaction scores calculated from the two data sets. By using all interactions, the Pearson coefficient is quite low (r=0.37). It's because the scores of non-interacted pairs are randomly around zero and a great part of gene pairs are non-interacted. We measured the correlation again by using only pairs which satisfy S>2.5 or S<-3. The correlation has been promoted significantly (r=0.65). In this case we could consider the two data sets are consistent.
