# Supplementary material for: Integrating multiple types of data to predict novel cell cycle-related genes
Source: BMC Syst Biol. 2011 Jun 20;5(Suppl 1):S9. doi: 10.1186/1752-0509-5-S1-S9 (PMC3121125; doi:10.1186/1752-0509-5-S1-S9)
Supplement: Additional file 5 — Cover rate when different TFs are selected This file can be viewed with Adobe Reader. [file 1752-0509-5-S1-S9-S5.pdf]

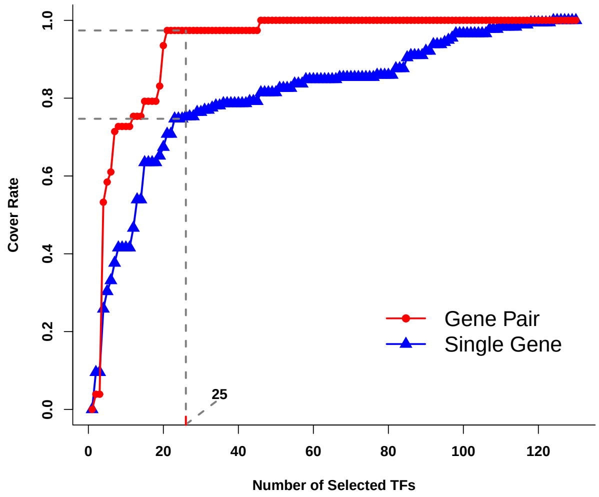

Figure S2 Cover rate when different TFs are selected. There are 232 genes in the PCCGs and KCCGs involved in the chip-chip data set (at least one TF can bind to them), and 77 interacted gene pairs can be simultaneously bound by the same TF. The figure shows when n TFs are selected, how many percent of the single genes and gene pairs can be covered. When top 25 TFs are selected, most of the 232 genes and 77 gene pairs (75% and 97%) could be covered, and when more TFs are selected the cover rate increases quite slowly. Hence we chose the top 25 TFs to construct the transcription network.
