# Supplementary material for: Integrating multiple types of data to predict novel cell cycle-related genes
Source: BMC Syst Biol. 2011 Jun 20;5(Suppl 1):S9. doi: 10.1186/1752-0509-5-S1-S9 (PMC3121125; doi:10.1186/1752-0509-5-S1-S9)
Supplement: Additional file 8 — Indirect TF-Target connection analysis This file can be viewed with Adobe Reader. [file 1752-0509-5-S1-S9-S8.pdf]

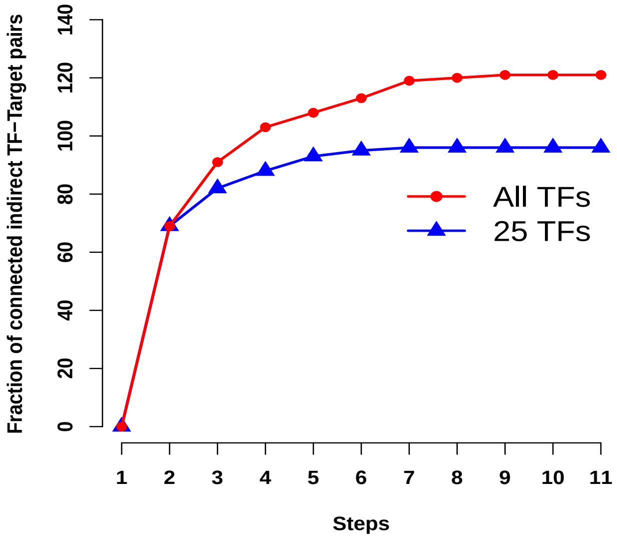

Figure S3: Indirect evidence supports the STs form a cooperative transcriptional network. 103 of 140 indirect interactions between the 25 TFs and their targets can be connected within 3 steps by using all TFs. If the 25 TFs are used, it can connect 85.4% (88/103) of these interactions in first 3 steps. This illustrates that the 25 TFs can form a well connected sub-network.
