# Supplementary figures and images for: Integrating multiple types of data to predict novel cell cycle-related genes
Source: BMC Syst Biol. 2011 Jun 20;5(Suppl 1):S9. doi: 10.1186/1752-0509-5-S1-S9 (PMC3121125; doi:10.1186/1752-0509-5-S1-S9)

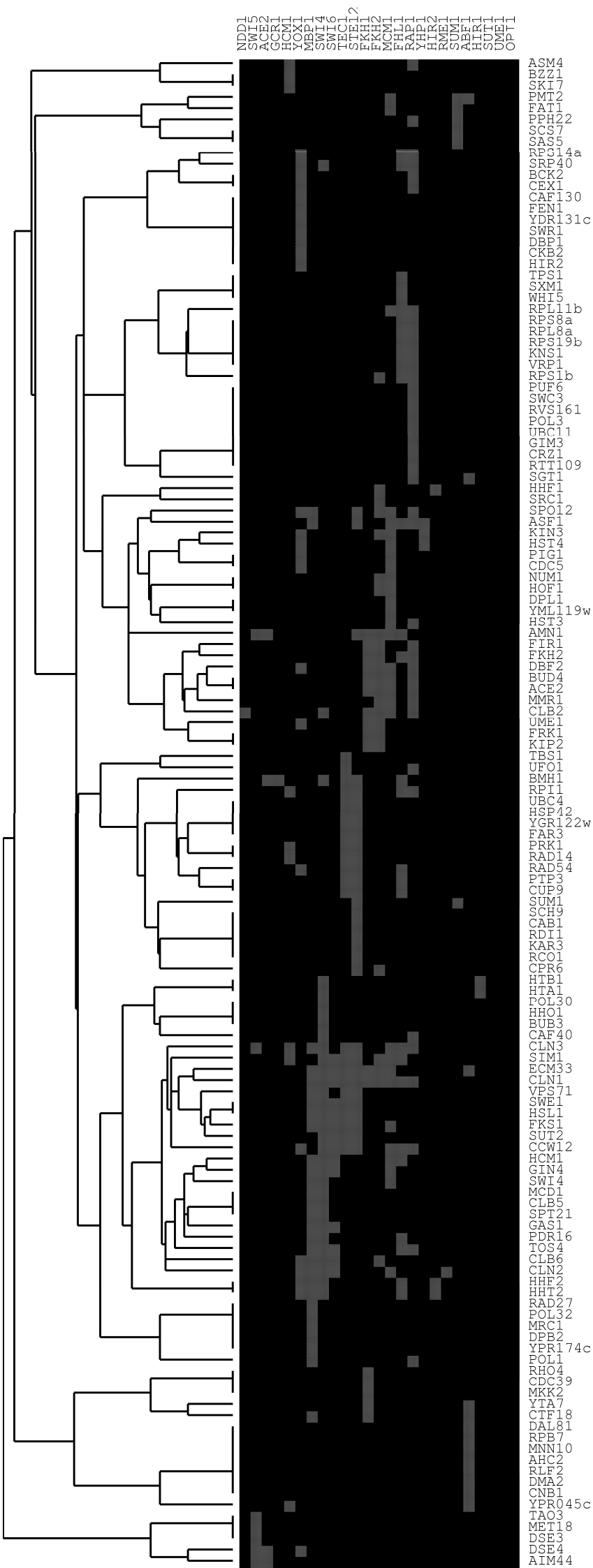

Supplement: Additional file 9 — Cluster results of transcriptional network This file can be viewed with Adobe Reader. This file can be viewed with Microsoft Excel Viewer. [file 1752-0509-5-S1-S9-S9.pdf]

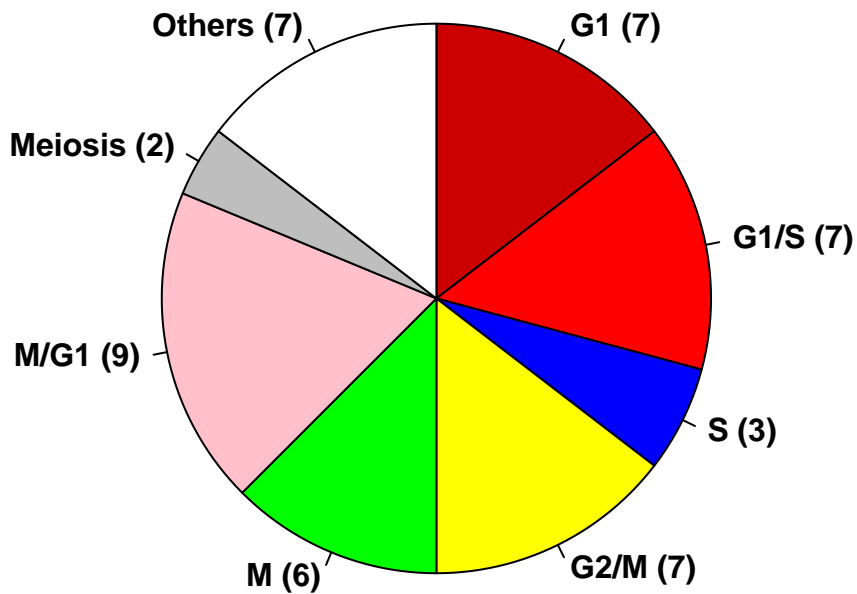

Supplement: Additional file 11 — Composition of the signaling E-MAP This file can be viewed with Adobe Reader. [file 1752-0509-5-S1-S9-S11.pdf]
